# Atlanta Academy of Medicine

**Published:** 1871-09

**Authors:** 


### ATLANTA ACADEMY OF MEDICINE.

This organization is rapidly becoming one of the recognized aids to the advancement of medical science, and is receiving contributions from medical men in various sections of the State. Among those recently received has been a package of cloth uterine tents, with accompanying explanation of advantages claimed for them, from Dr. V. H. Taliaferro, of Columbus, Ga. This, with a report of the discussion by the Academy upon the merits of these tents—in which Dr. Taliaferro participated while on a visit to this city—we had confidently calculated upon publishing in this number of the "Journal," but have been disappointed in receiving the manuscript, in consequence of the absence of the Academy Reporter from the city. We shall not undertake to report what are claimed to be the advantages of the cloth tents at present, but will publish the papers alluded to in the October number of the "Journal." We will only say, that Dr. Taliaferro made a most favorable impression upon the members of the Academy of Medicine by the efficient manner in which he sustained himself in the discussion of the merits of his tents, and strongly impressed the body with his familiarity with uterine diseases and their most advanced therapeutics.

